# Validation of operant social motivation paradigms using BTBR T+tf/J and C57BL/6J inbred mouse strains

**DOI:** 10.1002/brb3.273

**Published:** 2014-08-13

**Authors:** Loren Martin, Hannah Sample, Michael Gregg, Caleb Wood

**Affiliations:** Department of Graduate Psychology, Azusa Pacific University901 E Alosta Ave, Azusa, 91702, California

**Keywords:** Autism, behavior, mouse, open field, progressive ratio, three-chamber task, valence

## Abstract

**Background:**

As purported causal factors are identified for autism spectrum disorder (ASD), new assays are needed to better phenotype animal models designed to explore these factors. With recent evidence suggesting that deficits in social motivation are at the core of ASD behavior, the development of quantitative measures of social motivation is particularly important. The goal of our study was to develop and validate novel assays to quantitatively measure social motivation in mice.

**Methods:**

In order to test the validity of our paradigms, we compared the BTBR strain, with documented social deficits, to the prosocial C57BL/6J strain. Two novel conditioning paradigms were developed that allowed the test mouse to control access to a social partner. In the social motivation task, the test mice lever pressed for a social reward. The reward contingency was set on a progressive ratio of reinforcement and the number of lever presses achieved in the final trial of a testing session (breakpoint) was used as an index of social motivation. In the valence comparison task, motivation for a food reward was compared to a social reward. We also explored activity, social affiliation, and preference for social novelty through a series of tasks using an ANY-Maze video-tracking system in an open-field arena.

**Results:**

BTBR mice had significantly lower breakpoints in the social motivation paradigm than C57BL/6J mice. However, the valence comparison task revealed that BTBR mice also made significantly fewer lever presses for a food reward.

**Conclusions:**

The results of the conditioning paradigms suggest that the BTBR strain has an overall deficit in motivated behavior. Furthermore, the results of the open-field observations may suggest that social differences in the BTBR strain are anxiety induced.

## Introduction

In recent years, studies on the etiology of autism spectrum disorder (ASD) have identified several putative causal factors that are often explored through the use of a mouse model. Even though these models are usually built with genetic or environmental manipulations that have some degree of construct validity to ASD, the behavioral phenotype is also explored to provide face validity to the symptoms of ASD, and to establish structure–function relationships. Currently, however, more appropriate assays are needed to better phenotype these mice, particularly in regard to measuring social motivation (Silverman et al. 2010b). While social deficits have always been part of the core diagnostic features of autism, recent theorists have suggested that deficits in social motivation play a central role in the manifestation of ASD. This Social Motivation Theory of Autism posits that ASD symptoms are a consequence of social motivation deficits rather than a cause of disrupted social interest (Chevallier et al. 2012). Our goal therefore was to utilize operant paradigms involving social rewards to develop quantitative measures of social motivation for mouse models of ASD and other disorders of social pathology,. To our knowledge, only one previous study has attempted to measure nonsexual social motivation in mice using an operant paradigm but with limited success and without fully automated reinforcer delivery (Matthews et al. 2005).

We chose to compare the BTBR T+tf/J (BTBR) and C57BL/6J (B6) inbred mouse strains to test the validity of our operant social motivation paradigms. The BTBR T+tf/J (BTBR) strain was first suggested as a mouse model for ASD by Moy et al. (2007) following their screen of 10 inbred mouse strains for autistic-like behavior. Since this initial screen, numerous studies have confirmed reduced social behaviors in this strain, and have reported decreased reciprocal interactions, restricted exploratory behaviors, and unusual vocalizations, leading to its common use as a model to study ASD (e.g., McFarlane et al. 2008; Pearson et al. 2011; Scattoni et al. 2008, 2011; Silverman et al. 2010a). In addition to their face validity for multiple domains of ASD, the BTBR strain was chosen based on its commercial availability and reported reproducibility across laboratories. While we recognize that the lack of construct validity to ASD makes it a questionable model to study this disorder (Dodero et al. 2013), its consistently reported that social deficits in comparison to the B6 strain provided a means to validate our operant social motivation paradigms.

## Materials and Methods

### Test subjects

For the social motivation paradigm, three groups of nine male mice were tested: group-housed BTBR T+tf/J (BTBR) mice, group-housed C57BL/6J (B6) mice, and individually housed B6 mice. All test subjects were greater than 8 weeks of age at the time of testing and testing was conducted over a 2-month period for each mouse. The nine group-housed B6 mice and nine group-housed BTBR mice were further tested in a valence comparison paradigm aimed at distinguishing social from food motivation.

Two separate cohorts of male BTBR and B6 mice were used for the open-field experiments. The first cohorts of six BTBR and B6 mice were tested in the open field after the completion of social motivation testing and the acquisition of the ANY-maze software and open-field arena. The second cohorts consisted of nine male BTBR and 12 male B6 mice that were tested for comparison to the first cohorts.

A pool of age-matched B6 stimulus mice was used for both the open-field and Social Motivation experiments. All mice were housed in a vivarium with a set 14:10 h light:dark cycle in a climate-controlled setting with temperature maintained at 20°C. All testing was conducted during the light phase of the cycle. All mice were housed in groups of 2–4, with the exception of those otherwise specified for experimental purposes. Mice were housed in ventilated cages (OptiMICE; Animal Care Systems, Centennial, CO, USA) and given a pellet feed (Purina 5001) and water *ad libitum*. Additionally, all mice were identified via ear punches. All mice were treated in accordance with the NIH guidelines for the care and use of animals in research and all procedures were approved by the Azusa Pacific University Institutional Animal Care and Use Committee.

### Testing apparatuses for social motivation and valence comparison

The apparatuses used for the social motivation and valence comparison experiments were four-center channel modular shuttle boxes from Med Associates Inc. (model ENV-010MC; St. Albans, VT, USA). These shuttle boxes measured 44 × 17.3 cm and were made of Plexiglas and stainless steel. Each box was divided into two chambers (the test chamber and target chamber) each measuring approximately 22 × 17.3 cm. The dividing wall between these two chambers was fitted with an auto-guillotine door (model ENV-010B) that was programmed to open or close. A wire grid was positioned in front of the auto-guillotine door to keep mice from freely moving between chambers while also allowing social contact between mice. Mice levers (ENV-3010M; Med Associates) were placed in the right chamber (the test chamber) and were associated with either opening the guillotine door or a food reward, depending upon the testing paradigm. A food reward was dispensed via a liquid dipper (ENV-202M-S; Med Associates), which was located in the test chamber between the two mice levers. Each chamber had a metal grid floor fitted with an eight-channel I/R controller (model ENV-253C; Med Associates) to monitor activity across the chamber floor. The entire apparatus was enclosed within a melamine sound-attenuating cubicle (model ENV-016MD; Med Associates). The operant programs were run using the Control version 1.21 software from Campden Instruments using customized programs written in the laboratory.

### Social motivation paradigm

Test subjects from each group were first trained to associate lever pressing with the social reward through a manual shaping program and then tested in a fully automated progressive ratio program. Each mouse was tested in a single session each day, 7 days per week, and rotated through shuttle boxes via random assignment. Between each testing session, shuttle boxes were cleaned using 70% ethyl alcohol. Additionally, following the completion of testing each day, all equipment was cleaned using a disinfecting detergent.

#### Shaping

A stimulus mouse was placed in the target chamber and a test mouse was placed in the test chamber and trained to press the lever using the method of shaping or reinforcement of successive approximations to the desired operant response. Reinforcement consisted of opening the guillotine door for 30 sec, thereby allowing access to the stimulus mouse through the wire grid. During this shaping procedure reinforcement was delivered manually by an observer pressing a button programmed to control the guillotine door. Observations of the mouse in the testing apparatus were made with the use of a Microsoft Lifecam camera mounted above the test chamber and attached to the PC computer running the program software. Each mouse was trained in a series of 1-h sessions until they demonstrated at least 10 operant responses (lever presses) over three consecutive testing sessions. If mice did not reach this criterion after 30 daily training sessions, they were removed from the experiment. Stimulus mice were alternated during shaping so that a different mouse was used every other day.

#### Testing

A stimulus mouse was again placed in the target chamber and the test mouse placed in the test chamber. During testing, a pool of 10 stimulus mice was assigned to each test mouse so that stimulus mice were only repeated after 10 daily test sessions. Stimulus mice used during shaping were not used during testing. The guillotine door was programmed to open on a progressive ratio schedule of reinforcement so that the number of operant responses (lever presses) necessary to obtain the social reward of 15 sec access to the target mouse was arithmetically increase by a fixed rate of 3 each trial. Therefore, the amount of effort or work required to receive the same social reward increased across trials of each testing session (i.e., trial 1 = 3 lever presses, trial 2 = 6 lever presses, trial 3 = 9 lever presses, etc.). When the test mouse stopped responding for five consecutive minutes, the testing session ended and the last completed (reinforced) ratio was recorded as the breakpoint. This dependent measure was used as an index of social motivation. Each mouse was tested for 20 consecutive daily sessions.

### Valence comparison of social versus food reward

After all three groups of test mice completed all of the paradigms of experiment 2, these same mice were tested in a task designed to compare the valence of the social reward with that of a food reward. For this experiment, one lever was continually associated with a social reward while the adjacent was associated with a food reward. Test mice were assigned alternating lever associations so as to compensate for potential lever preferences among mice.

#### Training

As the mice had previously learned to lever press for a social reward, the focus of this training paradigm was to teach the mice to discriminate between the left and right levers through differential reinforcement. During this paradigm, a stimulus mouse was placed in the target chamber and a test mouse was placed in the test chamber. For half of the mice in each group, reinforcement of the left lever consisted of opening the guillotine door for 15 sec allowing access to the stimulus mouse through the wire grid. For these same mice, reinforcement of right lever presses consisted of 0.02 mL of evaporated milk sweetened with 0.2% sucrose solution presented for 15 sec by the liquid dipper into the food magazine. For the other half of the mice in each group, the lever/reward contingencies were reversed. In order to facilitate training of lever/reward contingencies, only one lever/reward contingency was active during each shaping session. Training consisted of six 1-h sessions that alternated between contingencies each day. Stimulus mice assigned to each test mouse were again alternated every other social reward session. Mice were maintained on their *ad libitum* chow diet during training and testing with the food reward. We have previously observed that food deprivation is unnecessary for operant conditioning in mice with the use of the evaporated milk and sucrose solution (L. Martin, H. Sample, and M. Gregg, unpublished observations).

#### Testing

The schedule of reinforcement was set at a fixed ratio of 3:1, so that each third lever press was reinforced, but only by its respective associated reward. The same pool of 10 stimulus mice was again assigned to the test mice for this paradigm following a 10-day rotation. Valence comparison testing sessions were 60 min in duration and sessions were carried out over 20 consecutive days. The primary dependent measure for this paradigm was the total number of lever presses.

### Testing apparatus for open-field experimentation

The apparatus used for this experiment was a Single Unit Open-Field Enclosure (San Diego Instruments, San Diego, CA, USA), composed of durable, high-density plastic. The dimensions were as follows: Base – 57.6 × 57.6 cm, wall height – 38 cm, inside chamber footprint – 50 × 50 cm. Two halogen desk lamps with 35W bulbs were placed on opposing sides of the arena and stood about 52 cm high from the base of the apparatus providing the sole source of light during testing. However, the lamps were angled at 45 degrees so that they did not directly shine upon the floor of the arena but rather in the middle of the arena wall opposite each lamp. The measured lux levels were 150 in the center of the arena and 150–200 around the perimeter. The apparatus was left open, clear of all objects, during acclimation and activity assessments. For the social choice and preference for social novelty paradigms, 2 black pencil cups (10.5 cm base diameter and 13.5 cm tall) were placed in opposing corners of the apparatus. A camera (Model TG3Z2910AFCS, Computer Optics Group, Commack, NY, USA) was placed centrally 78 cm over the enclosure and connected to a laptop running the ANY-maze video-tracking system. The video-tracking software was adjusted to track the center of gravity of the test mouse; movement of the stimulus mice was hidden by the black pencil cups and thus did not interfere with tracking. Movements were tracked in different virtual zones of the arena defined through the software program. A perimeter zone was defined 7 cm from the walls of the arena. A square center zone measuring 43 × 43 cm was defined within the perimeter. Perimeter zones were also defined 7 cm around each pencil cup on the floor of the arena available for exploration by the test mice.

### Open-field ANY-maze video-tracking assessments

The mice were tested in three different paradigms in an open-field arena using the ANY-maze video-tracking system. All test mice were acclimated to the arena, and stimulus mice were acclimated to the cups, for 10-min periods 24 h before testing. The arena was cleaned between testing sessions with 70% alcohol.

#### Open-field activity

The test mouse was placed in the clean, empty arena with the ANY-maze program set to track the mouse's movements. Each mouse was in the arena for 10 min and then removed.

#### Social choice

The test mouse was placed in the clean arena with a single stimulus mouse placed under a cup in either the top left or bottom right corners of the arena. The opposing cup was left empty. The placement of the stimulus mouse versus the empty cup was counterbalanced. Each mouse was in the arena for 10 min and then removed.

#### Preference for social novelty

The test mouse was placed in the clean arena with a familiar stimulus mouse under the cup in one corner of the testing arena and a novel mouse under the cup in the opposite corner. Each mouse was in the arena for 10 min then removed.

### Data analysis

All data were analyzed using SPSS version 17.0 or later. Dependent measures included breakpoint, total lever presses for the social motivation paradigm and food lever presses, social lever presses, food rewards, social rewards, and response duration (mean duration of a lever press) for the valence comparison paradigm. For the ANY-maze testing, dependent measures included total distance travelled, time spent in the center zone, time spent in the perimeter zone, and time spent in the zones are the pencil cups. Parametric statistical models including ANOVAs and independent samples *t*-tests were used as appropriate to compare dependent measures across the levels of the independent variables. Repeated measures analysis of variance was used to analyze within-subjects differences across testing conditions. Appropriate post hoc analyses were conducted based upon the data.

## Results

### Social motivation paradigm

**Figure 1:**
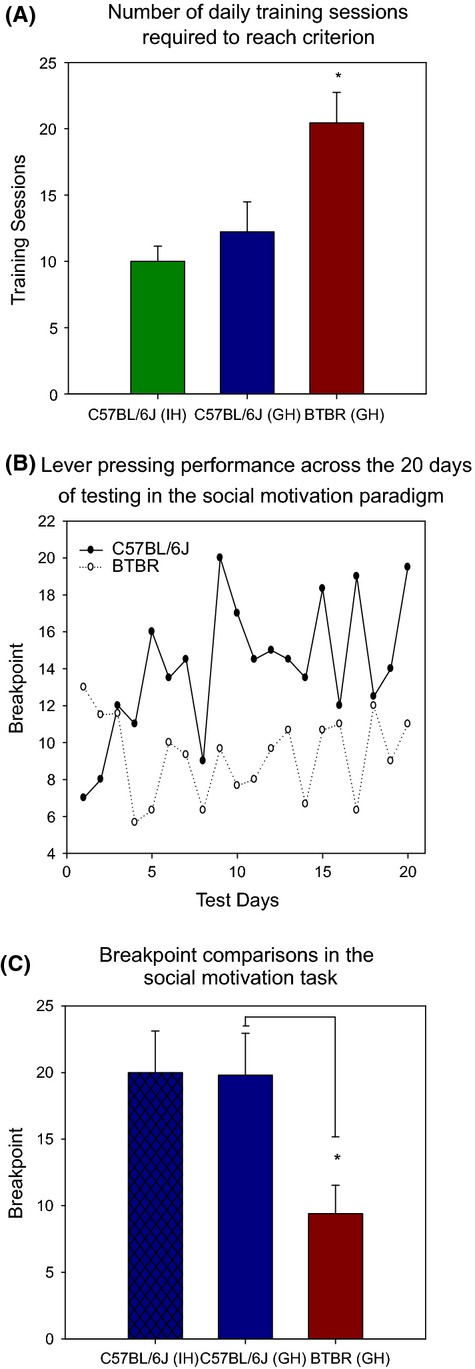
Results from the social motivation testing paradigm. (A) The number of daily training sessions required to reach criterion of at least 10 operant responses over three consecutive days. Only nine of 17 BTBR mice reached criterion while all B6 mice successfully learned the task regardless of housing condition. The BTBR mice that did learn to press for the social reward required significantly more training sessions to do so than the housing-matched B6 mice. (B) Mean breakpoint of group-housed BTBR and B6 mice across testing sessions of the social motivation paradigm. Asymptotic performance was observed soon after mice moved from the training to the testing phase of the paradigm. (C) Mean breakpoint of individually housed B6 mice (*n* = 9), group-housed B6 mice (*n* = 9), and group-housed BTBR mice (*n* = 9) across the last 10 days of testing in the social motivation task. There was no significant difference between IH and GH B6 mice, however, there was a significant difference between the GH B6 versus BTBR mice. In all figures, *indicates significant results.

Three groups of mice were tested in the social motivation paradigm consisting of nine mice per group: group-housed BTBR mice, group-housed B6 mice, and individually housed B6 mice. Comparisons were made between the group-housed mouse strains and between housing conditions for the B6 strain. All B6 mice, regardless of housing condition, successfully learned to associate lever pressing with the social reward. For the BTBR strain, only nine of 17 mice successfully learned to associate lever pressing with the social reward after 30 daily training sessions. The nine BTBR mice that did successfully learn the task required a mean of 20.44 training sessions (SD = 6.86) which was significantly more than the group-housed B6 mean of 12.22 (SD = 6.80; *t* = −2.555, df = 16, *P* = 0.021; Fig. 1A). These results are consistent with our observations of food-deprived BTBR mice in other operant tasks involving a food reward and thus do not appear to be specific to the social reward (L. Martin, H. Sample, and M. Gregg, unpublished observations).

After successfully learning the task, testing in the social motivation paradigm was carried out over 20 daily sessions. Breakpoint results indicate that asymptotic performance was achieved very early in the testing phase by most of the mice (Fig. 1B). However, to ensure performance stabilization, independent sample *t*-tests were only carried out using the mean breakpoint over the last 10 days of testing for each group. As shown in Fig. 1C, the mean breakpoint of the BTBR mice (*M* = 9.40. SD = 6.39) was significantly lower than that of the B6 mice (*M* = 19.8, SD = 9.43) that were also group-housed (*t* = 2.741, df = 16, *P* = 0.015). There was no difference observed between the group-housed and individually housed (*M* = 20.0, SD = 9.37) B6 mice (*t* = 0.043, df = 16, *P* = 0.967).

### Valence comparison paradigm

**Figure 2:**
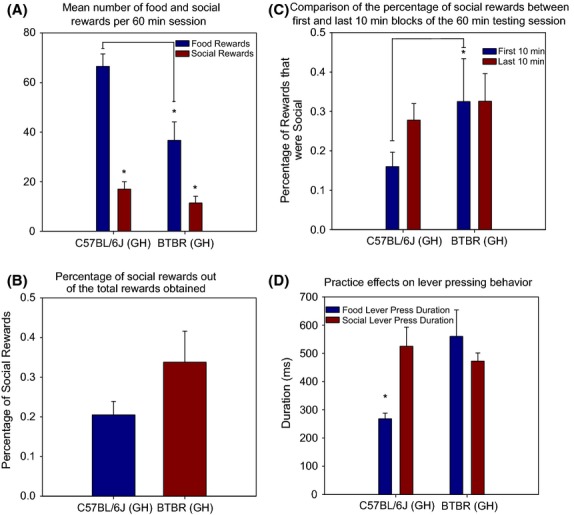
Results from the valence comparison testing paradigm. (A) The mean number of food rewards and social rewards for the group-housed B6 and BTBR mice across the last 10 sessions of the valence comparison paradigm. There were significantly more food rewards obtained than social rewards for both mouse groups. However, the BTBR mice obtained significantly fewer food rewards than the B6 mice and also the fewest number of social rewards (data not significant). (B) There were no significant differences in the percentage of social rewards between the mouse groups. (C) The percentage of social rewards significantly increased in the B6 mice. However, for the BTBR mice, there was no difference in the percentage of social rewards across testing. (D) Paired sample *t*-tests showed food lever presses (regardless of whether it was assigned to the left or right lever) to be significantly faster, and thus more efficient than social lever presses in the B6 mice but not the BTBR mice.

The group-housed BTBR and B6 mice were further tested in the valence comparison paradigm designed to compare social and food motivation (see Movie S1). As expected, paired samples *t*-tests demonstrated that both mouse strains had significantly more lever presses for food rewards than social rewards (BTBR: *t* = 3.551, df = 8, *P* = 0.007; B6: *t* = 9.478, df = 8, *P* < 0.001; Fig. 2A). The percentage of the total rewards that were social rewards was calculated for each mouse to make comparisons between strains. Independent sample *t*-tests revealed no significant differences between strains in the social reward percentage (*t* = −1.565, df = 10.936, *P* = 0.146; Fig. 2B). While the BTBR mice demonstrated fewer lever presses for a social reward (*M* = 34.17, SD = 24.28) than the B6 mice (*M* = 51.00, SD = 26.84), the difference was not significant (*t* = 1.395, df = 16, *P* = 0.182). Surprisingly, however, the BTBR mice did have significantly fewer lever presses for food rewards (*M* = 110.07, SD = 67.00) than the B6 mice (*M* = 199.57, SD = 45.24; *t* = 3.321, df = 16, *P* = 0.004; Fig. 2A).

In order to assess the effects of reward satiation on lever-pressing behavior, comparisons were made between the first and last 10 min blocks of the 60-min testing sessions of the valence comparison paradigm. Paired sample *t*-tests showed that the B6 mice had significantly fewer food rewards (*t* = 7.871, df = 8, *P* < 0.001) but not social rewards (*t* = 1.866, df = 8, *P* = 0.099) in the last 10-min block compared to the first 10-min block (Fig. 2C). Thus, the percentage of total rewards that were social significantly increased from 16 to 27.8% across these testing blocks (*t* = −4.585, df = 8, *P* = 0.002). The BTBR mice did not have any significant differences in lever-pressing behavior between the first and last 10-min blocks of the testing sessions (Food Rewards: *t* = 1.118, df = 8, *P* = 0.296; Social Rewards: *t* = 0.915, df = 8, *P* = 0.387; Percentage Social: *t* = −0.003, df = 8, *P* = 0.998).

As shown in Fig. 2D, additional analyses of lever-pressing behavior involving the amount of time the lever was depressed (i.e., lever-press duration) was conducted on the test mice. Paired sample *t*-tests showed lever presses for a food reward (regardless of whether it was assigned to the left or right lever) to be significantly faster, and thus more efficient, than lever presses for a social reward in the B6 mice but not the BTBR mice (B6: *t* = 5.137, df = 8, *P* = 0.001; BTBR: *t* = 0.011, df = 4, *P* = 0.991). Comparisons between strains showed that lever-press durations for a food reward were significantly faster for the B6 mice (*t* = −3.035, df = 8.737, *P* = 0.015) but there was no significant difference in lever-press durations for a social reward (*t* = 0.718, df = 16, *P* = 0.483).

### Open-field assessments

**Figure 3:**
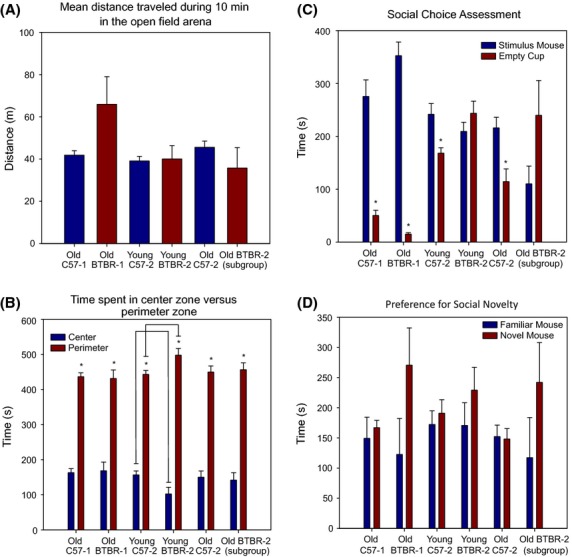
Results from the ANY-maze video-tracking assessments in the open-field arena. (A) Mean distance travelled during 10 min in the open-field arena. Regardless of cohort age or strain, there were no significant differences in the total distance travelled during the activity assessment. (B) Time spent in center zone versus perimeter zone during 10 min in the open-field arena. All cohorts of test mice from both strains demonstrated a significant preference for the perimeter over the center of the arena. The younger cohort of BTBR mice exhibited significantly more thigmotaxis than the younger cohort of B6 mice. There were no significant differences in thigmotaxis observed between the older cohorts of mice. (C) Social choice assessment for 10 min in the open-field arena. The first cohorts of older B6 and BTBR mice spent significantly more time exploring the stimulus mouse over the empty cup. The younger cohort of B6 mice demonstrated a similar result but the younger cohort of BTBR mice did not show a preference for the cup containing the stimulus mouse. They also differed from the older BTBR mice in the amount of time spent in both the stimulus mouse and empty cup zones. A follow-up study on the second cohorts of B6 and available BTBR mice several months later revealed that the B6 mice maintained their social preference and the subgroup of available BTBR mice maintained a lack of preference for the stimulus mouse zone. (D) Preference for social novelty assessment for 10 min in the open-field arena. Regardless of strain or age at the time of testing, there were no significant differences in the time spent in the familiar mouse zone compared to the time spent in the novel mouse zone.

The ANY-maze video-tracking software and associated equipment were acquired following the completion of testing in the operant paradigms. As such, only a subset of mice that completed testing in the operant paradigms was also available for testing in the open-field experiments (6 mice per genotype) and they were all between 32 and 40 weeks of age at the time of testing. Mice were tracked in 10-min blocks to measure activity, social choice, and preference for social novelty. Results from the 10-min activity assessment in the empty open-field arena showed that there were no significant differences in total distance travelled (*t* = 1.817, df = 5.266, *P* = 0.126; Fig. 3A). As shown in Fig. 3B, both mouse strains spent significantly more of their time in the perimeter (BTBR: *M* = 431.32 sec, SD = 60.05; B6: *M* = 436.65 sec, SD = 26.97) versus the center (BTBR: *M* = 168.67 sec, SD = 60.07, *t* = 5.356, df = 5, *P* = 0.003; B6: *M* = 163.35 sec, SD = 26.97, *t* = 12.409, df = 5, *P* < 0.001) of the arena but the amount of time spent in each of these zones was similar between the strains. As shown in Fig. 3C, both strains spent significantly more time in the zone around the stimulus mouse than the zone around the empty cup during the 10-min social choice assessment (BTBR: *t* = 12.281, df = 5, *P* < 0.001; B6: *t* = 6.642, df = 5, *P* = 0.001). Interestingly, the BTBR mice spent significantly less time in the empty cup zone than the B6 mice (*t* = −3.387, df = 5.701, *P* = 0.016) and demonstrated a trend to spend more time in the stimulus mouse zone, even with the small sample size (*t* = 1.903, df = 10, *P* = 0.086). Figure 3D shows the results from the 10-min preference for social novelty assessment. Paired samples *t*-tests revealed that neither mouse strain showed a preference for a novel mouse over a familiar mouse (BTBR: *t* = 1.260, df = 5, *P* = 0.263; B6: *t* = 0.414, df = 5, *P* = 0.696). Additionally, there were no significant differences in the amount of time that each strain spent in each respective zone (Novel Mouse: *t* = 1.643, df = 5.38, *P* = 0.157; Familiar Mouse: *t* = −0.387, df = 10, *P* = 0.707).

Due to the age of the mice at the time of testing in the open-field assessments, testing was repeated on younger cohorts of 9 BTBR and 12 B6 mice that were between 7 and 12 weeks of age (mean age of 9.14 and 10.19, respectively). Similar to the older cohorts, there was no significant difference in total distance travelled (*t* = −0.158, df = 19, *P* = 0.876; Fig. 3A) and both mouse strains again spent significantly more time in the perimeter than the center of the arena (BTBR: *t* = 10.357, df = 8, *P* < 0.001; B6: *t* = 12.825, df = 11, *P* < 0.001; Fig. 3B). However, the difference in the amount of time spent in the perimeter versus the center of the arena was significant between the mouse strains (*t* = −2.609, df = 19, *P* = 0.017). While this finding was only marginally significant after applying a bonferroni correction, it was confirmed by comparing the group of nine young BTBR mice to a second cohort of eight young B6 mice (*t* = −3.475, df = 15, *P* = 0.003; data not shown). The lack of a difference in the older mouse cohorts seemed to be due to an age-related effect in the BTBR strain as there was no difference between young and old B6 on this measure (*t* = 0.370, df = 16, *P* = 0.716), but a trend for a difference between young and old BTBR mice (*t* = 2.159, df = 13, *P* = 0.050).

In the social choice task, the younger B6 cohort spent significantly more time in the stimulus mouse zone than the empty cup zone (*t* = 3.497, df = 11, *P* = 0.005) similar to the cohort of older B6 mice (see Fig. 3C). However, the younger BTBR cohort did not show this preference for social contact (*t* = −0.901, df = 8, *P* = 0.394) in contrast to their older counterparts. Indeed, comparisons between the older and younger BTBR cohorts revealed that the differences in the amount of time spent in the stimulus mouse zones and empty cup zones were each highly significant (Stimulus Mouse: *t* = 4.804, df = 13, *P* < 0.001; Empty Cup: *t* = −9.959, df = 8.216, *P* < 0.001) with the older BTBR mice demonstrating a clear preference for social engagement (Fig. 3C). The results from the preference for social novelty task were similar to those found in the older cohorts in that neither strain demonstrated a significant preference for the novel mouse over the familiar mouse (BTBR: *t* = 0.832, df = 8, *P* = 0.430; B6: *t* = 0.555, df = 11, *P* = 0.590; Fig. 3D).

We did a follow-up study on the mice from the younger cohorts by repeating the ANY-maze testing several months later. All of the B6 mice but only four of the BTBR mice were still available for follow-up testing and they ranged in age from 42 to 52 weeks at the time of testing (BTBR mean = 42.14 and B6 mean = 49.86). Once again, the BTBR mice spent significantly more time in the perimeter than the center of the arena (*t* = 7.739, df = 3, *P* = 0.004) and these results were very similar to the activity results observed when they were young (perimeter: *t* = −0.142, df = 3, *P* = 0.896; center: *t* = 0.188, df = 3, *P* = 0.863; Fig. 3B). Results from the social choice assay were also very similar to the results from the earlier assessment of these same mice (see Fig. 3C). The BTBR mice spent a mean of 239.75 sec (SD = 131.58) in the empty cup zone compared to 110.10 sec (SD = 67.43) in the stimulus mouse zone, and similar to their earlier results, these times were not significantly different from each other (*t* = 1.359, df = 3, *P* = 0.267). On the other hand, the B6 mice again showed a preference for the stimulus mouse over the empty cup (*t* = −2.533, df = 11, *P* = 0.028). Paired samples *t*-tests across testing timepoints for all of the measures did not reveal any significant differences (see Fig. 3C). Also, as with the previous preference for social novelty tests, there were no differences in the amount of time spent in the novel or familiar mouse zones for either mouse strain (see Fig. 3D).

## Discussion

### Differences in learning ability between strains

A comparison of the training sessions between BTBR and B6 strains demonstrated evidence for a learning impairment in the BTBR strain. Approximately half of the BTBR mice did not learn to lever press for a social reward after 30 days of daily 1-h shaping sessions. In addition, those mice that did successfully learn to associate lever pressing with a social reward took significantly longer to reach the learning criterion than the B6 strain. These results do not seem to be specific to the social reward as we have observed similar results when training food-deprived BTBR mice to lever press for a food reward in a different study. The learning deficits do seem to be specific to these learned associations, however, as other laboratories have reported normal spatial discrimination in the Morris water maze (Moy et al. 2007; Yang et al. 2012) and a 100% accurate reversal learning task as well as the acquisition phase of a probabilistic reversal learning task (Amodeo et al. 2012). BTBR mice did take significantly longer to reach criterion in the probabilistic reversal learning task though, leading the authors to conclude that the increased level of difficulty of this task resulted in their learning deficits. This may reflect a similar impairment that we observed in operant conditioning. Perhaps BTBR mice can readily make simple associations but struggle to make more complex associations such as manipulating an object to gain a reward.

The inconsistency in learning within this strain is a concern as it demonstrates some of the heterogeneity of this inbred mouse. The BTBR strain is known to have agenesis of the corpus callosum and anatomical studies of this strain have consistently shown a complete absence of this structure (Wahlsten et al. 2003; Dodero et al. 2013; Ellegood et al. 2013). Indeed, in our own histological sampling of these mice we have confirmed a complete absence of the corpus callosum (L. Martin, H. Sample, and M. Gregg, unpublished observations). The callosal fibers of the hippocampal commissure are also maldeveloped in the BTBR strain. However, this phenotype appears to be much more variable (Wahlsten et al. 2003). We are therefore currently exploring the possible association between the size of the hippocampal commissure and associative learning in these mice.

### Reduced motivation of BTBR mice

For those BTBR mice that were able to learn the association between lever pressing and the social reward, the results of the social motivation paradigm demonstrated a significant reduction in lever pressing compared to the B6 mice. This finding is consistent with previous studies suggesting reduced sociability of the BTBR strain (Bolivar et al. 2007; McFarlane et al. 2008). However, the results from the valence comparison paradigm showed that the BTBR mice also demonstrated reduced lever pressing for a food reward compared to the B6 strain. In addition, there was no difference in the percentage of social rewards between the strains. Taken together, these results suggest that the BTBR mice do not have a selective deficit in social motivation but rather exhibit a more generalized deficit in motivated behavior.

### Validation of the social motivation tasks

Overall, the social motivation tasks proved to be very promising tools for the phenotyping of social behavior in mouse models. In the social reward paradigm, the BTBR strain demonstrated the expected decrease in lever-pressing behavior for a social reward compared to the B6 mice. However, the valence comparison paradigm showed that this reduction in lever-pressing behavior was not specific to the social nature of the reward. These combined results demonstrate the power and utility of these sophisticated means of measuring social motivation in mice. Validation that these tasks are measuring motivated behavior comes from the comparison across testing blocks in the valance comparison paradigm. The B6 mice significantly reduced their lever-pressing behavior for a food reward but not a social reward between the first and last 10-min blocks of the testing sessions. As the food rewards outnumbered the social rewards by a little more than a 5:1 ratio during the first 10-min block but less than a 2:1 ratio during the last 10-min block, it is apparent that the motivation for the B6 mice to work for the food reward was reduced as they became satiated with the reward (Fig. 2C).

It is important to note that observations made during manual shaping of the social motivation task showed that both the test and stimulus mice converged on the wire grid in front of the open guillotine door and engaged in social interactions during the social reward phase (see Movie S1), However, it would be ideal for future studies using these tasks to verify the presence of both mice at the door during social reward with sensors and quantify the amount of time that each mouse spends in social engagement during the 15-sec reward period.

### Measures of motor performance in the social motivation tasks

The lever-press duration is an important measure in the social motivation tasks as well. If a particular mouse strain demonstrates a significant increase in lever-press duration, regardless of the reward, it may indicate the presence of a motor impairment that could impact motivation. For example, we previously demonstrated that the lurcher mutant, an inbred mouse strain with obvious motor ataxia, had much longer lever-press durations in an operant task than wild-type siblings. This finding suggested that lever pressing demanded much more effort from the lurcher mice than the wild-type mice which likely explained their overall reduction in lever-pressing behavior (Martin et al. 2010). The fact that the BTBR mice had similar lever-press durations to the B6 mice when pressing for a social reward indicates that their reduction in lever-pressing behavior is not related to a motor deficit. However, it is interesting to point out how much more efficient the B6 mice became when lever pressing for a food reward (Fig. 2D).

### Open-field assessments

Results from the open-field assessments using ANY-maze video tracking showed that both strains travelled similar distances and demonstrated thigmotaxis in the open-field arena. However, while the first cohort of BTBR mice spent similar amounts of time to their age-matched B6 counterparts in the perimeter and center zones, the second cohort of BTBR mice demonstrated a significantly greater difference in time spent in the perimeter versus the center than their age-matched B6 counterparts and this difference was confirmed through a comparison with another age-matched cohort of B6 mice. As the ratio of time spent in the perimeter versus center of an open-field arena is considered a measure of anxiety, the greater thigmotaxis observed in the younger BTBR cohort is consistent with other studies reporting heightened anxiety in the BTBR strain (Benno et al. 2009; Frye and Llaneza 2010; Pobbe et al. 2011).

In the social choice task, the first cohort of BTBR mice showed a clear preference for the stimulus mouse over the empty cup, even more so than the age-matched B6 cohort, and counter to previous studies (McFarlane et al., 2008, Pobbe et al. 2011; Yang et al. 2011). Given the older age of the BTBR mice at the time of testing, we hypothesized that the BTBR mice may become more social as they age. We therefore tested a second cohort of BTBR mice at a younger age and found that they did not show the preference for the stimulus mouse as their age-matched B6 counterparts. However, besides the age difference in the two BTBR cohorts tested, there were also differences in handling and social exposure. The older BTBR mice had completed the social motivation tasks prior to the open-field testing and thus were handled daily and exposed to novel stimulus mice for 8–12 weeks. We therefore decided to test the younger cohort of BTBR mice again after they had aged. Unfortunately, when this decision was made five of the nine BTBR mice from the second cohort had already been euthanized. The remaining four mice were tested in the open-field tasks a second time, approximately 33 weeks following the first round of tests.

The results from this subgroup of BTBR mice were very similar to the first round of testing suggesting that age does not account for the differences in sociality between the first and second BTBR cohorts. As these four mice were not tested in any other paradigms, they were not exposed to the daily handling and social encounters with novel mice that the first cohort of BTBR mice experienced. These environmental differences may potentially explain the differences in sociality between these cohorts, especially given the anxiolytic effects that daily handling and social exposure may have on the mice. Indeed, BTBR mice have been reported to have both general and social anxiety (Pobbe et al. 2011), and housing BTBR mice with the more sociable B6 strain has been shown to rescue the social deficits of BTBR mice (Yang et al. 2011). However, given the small sample size of the aged BTBR mice in the second cohort, a clear interpretation for these sociality differences cannot be made without further testing. Differences in sociality between BTBR cohorts may simply be due to genetic drift within the strain.

The preference for social novelty testing did not yield the typical results observed in other studies in which both mouse strains indicate a preference for a novel mouse. However, this test is highly variable across strains and has been shown to yield conflicting results within strains (DBA/2J and AKR/J), even in the same laboratory (Moy et al. 2007, 2008). Nevertheless, the variation in our results may be due to methodological differences between studies as these previous studies employed a three-chambered apparatus, while we utilized an open-field arena.

## Conclusion

Overall, this study demonstrates the feasibility and validity of the social motivation operant tasks. The social motivation paradigm provides a quantitative measure of social motivation which in this case showed that the BTBR strain had reduced social motivation in comparison to the B6 strain, consistent with previous studies on the sociability of these strains. In addition, the valence comparison paradigm provides an important control for deficits in generalized motivation which in this case demonstrated that the motivation deficits observed in the BTBR strain were not specific to social rewards. The fixed session length of the valence comparison paradigm provides researchers with the ability to track motivated behavior over time. These data are also very valuable as it enables researchers to measure changes in motivated behavior over time associated with satiety. Other means of measuring social behavior in mice, including social choice, preference for social novelty, social transmission of food preference, and social place preference tasks among others, require very little effort from the test mouse. In our operant social motivation task, test mice are required to work for a social reward and the amount of work required for a single social encounter increases each trial providing a quantitative measure of effort and an index of social motivation.

While the results of the social motivation paradigm are consistent with previous studies on the sociability of the BTBR strain, the findings from the valence comparison paradigm provide a cautionary tale regarding the use of the BTBR strain as a model for autism. A PubMed search reveals that there are no less than 75 publications to date that connect the BTBR strain to autism in some way. However, many of these studies seem to be focused on seeking confirming evidence for the face validity of BTBR behaviors to autism. Indeed, as a whole, the reported phenotypes of the BTBR strain are quite unique from most cases of autism and have led to its use in the study of a number of diseases. In the absence of any construct validity to autism, that is, any causal factors, autism researchers would be best advised to consider other mouse model options.

## Supporting Information

**Movie S1.** Representative C57BL/6J mouse demonstrating lever-pressing behavior for a social reward (first) and then a food reward (second) during the valence comparison paradigm. Note that the food receptacle light was turned on during food presentation for video purposes only. It was not programmed to turn on during testing.
